# Competing interests, clashing ideas and institutionalizing influence: insights into the political economy of malaria control from seven African countries

**DOI:** 10.1093/heapol/czaa166

**Published:** 2020-12-14

**Authors:** Justin Parkhurst, Ludovica Ghilardi, Jayne Webster, Robert W Snow, Caroline A Lynch

**Affiliations:** 1 Department of Health Policy, London School of Economics and Political Science, Houghton Street, London WC2A 2AE, UK; 2 London School of Hygiene and Tropical Medicine, Keppel Street, London WC1E 7HT, UK; 3 Kenya Medical Research Institute-Wellcome Trust Research Programme, P.O. Box 43640-00100, Nairobi, Kenya

**Keywords:** Political economy, malaria control, health policy, ideas, interests, institutions

## Abstract

This article explores how malaria control in sub-Saharan Africa is shaped in important ways by political and economic considerations within the contexts of aid-recipient nations and the global health community. Malaria control is often assumed to be a technically driven exercise: the remit of public health experts and epidemiologists who utilize available data to select the most effective package of activities given available resources. Yet research conducted with national and international stakeholders shows how the realities of malaria control decision-making are often more nuanced. Hegemonic ideas and interests of global actors, as well as the national and global institutional arrangements through which malaria control is funded and implemented, can all influence how national actors respond to malaria. Results from qualitative interviews in seven malaria-endemic countries indicate that malaria decision-making is constrained or directed by multiple competing objectives, including a need to balance overarching global goals with local realities, as well as a need for National Malaria Control Programmes to manage and coordinate a range of non-state stakeholders who may divide up regions and tasks within countries. Finally, beyond the influence that political and economic concerns have over programmatic decisions and action, our analysis further finds that malaria control efforts have institutionalized systems, structures and processes that may have implications for local capacity development.

**KEY MESSAGES**

- National malaria control policies and actions are driven by more than simply technocratic reviews of evidence for specific intervention strategies.
- The political economy of malaria control is influenced by: the interests of influential funders and implementing stakeholders; dominant ideas in the global malaria community; and institutional arrangements shaping national policy and practice.
- Malaria funding concentrated into a small number of lenders, but a large number of implementers, may restrict national control over planning and action.

## Introduction

Political scientists studying health policy have noted that while public health planning may commonly be framed as a rational technocratic exercise in problem-solving, the realities of policymaking rarely reflect this public health ideal (Bernier and Clavier, 2011). Rather, authors have explained that there is a need to engage with a number of political and economic factors to understand why and how health policy decisions and outcomes come about outside the evaluation of technical evidence alone—factors including aspects of stakeholders and networks, power and influence, and governance or institutional contexts (de Leeuw *et al.*, 2014; Gilson *et al.*, 2018). Although those involved in health policy decisions are well aware that they are acting within a number of political constraints, they do not necessarily work to define or analyse them explicitly.

Malaria control represents one such example of a global health priority that is commonly conceptualized as a technical public health issue. The World Health Organization (WHO) is the leading international actor providing advice and information on malaria control, and identifies three principle strategies for malaria prevention: use of insecticide-treated mosquito nets, indoor residual spraying (of insecticide) or chemo-prophylaxis (administering anti-malarial drugs to key populations at particular times before exposure to prevent malaria illness). The WHO further recommends malaria treatment options through confirmed diagnosis and treatment in the health system, or via community-based management strategies for under-resourced areas (WHO, 2017). The overarching WHO guideline document is itself titled the ‘Global Technical Strategy’ (WHO, 2015)—with such documents rarely including explicit consideration of political aspects of malaria control (although there may be mention of a need for political will). Decision-making for malaria control is therefore typically conceptualized as an exercise involving review of evidence and modelling to inform decisions between combinations of interventions and strategies to maximize the chances of control or, indeed, eventual elimination of malaria (Hemingway *et al.*, 2016; Tediosi *et al.*, 2017).

While other global health issues have been the subject of significant political analysis [HIV/AIDS being a notable example (Seckinelgin, 2007; Poku and Whiteside, 2017)], malaria control discussions often remain in this technocratic framing. It may be that malaria is less frequently a feature of electoral politics in comparison to apparently controvertial issues such as HIV/AIDS, abortion or vaccination. However, there have been some examples of political controversy around national malaria decisions explored in the academic literature (see Tesfazghi *et al.*, 2016), and so it may be that local politicization has simply not been the topic of analysis often, even if it does exist. The fact that little has been written focused at national levels to explore the dynamics of decision-making and implementation for such an important public health issue, however, provides an important justification for more exploratory politically informed empirical work on this topic.

Indeed, in many ways, malaria control may be typical of a range of global health issues for which there are established systems of international donor and technical expert activity working to shape decision-making and implementation, often without explicitly critical political lenses being applied. Yet the activity of both donors and global expert bodies has elsewhere been shown to bring their own set of political economy concerns in the context of low- and middle-income settings. For example, the roles of international actors and donor bodies have been critiqued in terms of how they may shape local agendas (Okuonzi and Macrae, 1995). The power and influence of global expertise have been highlighted as exercising power without necessarily considering accountability concerns (Shiffman, 2014). And a number of works in the health and development sector have shown how donor efforts may not account sufficiently for local contexts, potentially imposing outside ideas and problem constructions on recipient nations (Ferguson, 1994; Honig and Gulrajani, 2018).

It thus remains important to understand the political realities of decision-making for health policy issues that may typically be framed as technical exercises in decision-making, but for which local and international contexts may play out in yet unexplored ways to shape intervention choices and activities implemented to achieve health goals. This paper arose from research nested within an explicitly technically oriented programme of work—the LINK-Data for Decision-making programme. That programme supported 13 high-prevalence countries to develop modelled malaria prevalence maps and epidemiological profiles—working under the theory that providing National Malaria Control Programmes (NMCPs) with improved epidemiological data (more recent and more specific maps of the epidemiological context) would lead to more effective and efficient planning and resource allocation at a country level [mapping methods can be found in Noor *et al.* (2014) and Snow and Noor (2015)].

The LINK programme was specifically one that aimed to provide evidence that could be useful to inform decision-making by allowing decision-makers to target or prioritize particular geographic areas and sets of interventions based on more up-to-date and more geographically specific knowledge of the local epidemiological situation. Yet while the technocratic instincts of the public health community may often portray data and evidence in a depoliticized fashion [as a technical tool that simply needs to be ‘used’ or ‘taken up’ (Oliver *et al.*, 2014)], public policy analyses of evidence use within health policymaking have found that key political elements such as how problems are framed, the interests of policy stakeholders, or institutional arrangements governing decision-making can all play important roles in shaping when, how or why certain forms of evidence are utilized (Parkhurst *et al.*, 2018).

Recognizing these realities, the LINK programme identified an opportunity to explore more directly the political and economic factors that were influencing malaria decision-making across a set of African countries being provided with data and evidence intended to be useful for planning. This aimed to provide insights about decision-making typically excluded from programmes providing data for technical decision-making support, and further help contribute to the broader understanding of malaria decision-making. This paper provides results from analyses of qualitative interviews conducted in LINK partner countries which enabled critical reflection on how and why particular malaria control decisions were made outside of the simple technical review of data and evidence alone. A broad and exploratory approach was taken, given the lack of knowledge around malaria control specifically, combined with a recognition of a large number of potential political concepts that might provide insights into decision-making and implementation choices.

## Background

Malaria remains one of the most important infectious diseases globally, remaining in the top 10 causes of early death according to the 2017 Global Burden of Disease study (IHME, 2018). The World Health Organization (WHO, 2019) has recently reported that progress in reducing malaria is now felt to be stalling, with 228 million malaria cases estimated in 2018. In that year, 19 countries accounted for 85% of the nearly half-million global malaria deaths—with 18 of these countries located in sub-Saharan Africa. The burden of the illness also primarily falls on the young, with approximately two-thirds (67%) of all malaria deaths occurring in children under 5 (WHO, 2019).

However, the bulk of malaria financing is controlled by a small number of actors. In 2018, national contributions from malaria-endemic countries represented only 30.5% of total financing. The USA, however, provided 37.3% of global malaria funding, and the UK 9.2% in that year—with around half of international funding channelled through the Global Fund for HIV, Tuberculosis and Malaria (the Global Fund) (WHO, 2019). This raises important concerns about the influence and role of international donors and non-state stakeholders in shaping national policy and programme outcomes.

Ultimately, malaria control responsibility falls to national governments. At the country level, NMCPs are the key local agency involved in planning and decision-making on the ground. They are typically officially mandated parts of, or delegates of, Ministries of Health (MoH) —expected to use available evidence to make strategic planning decisions on malaria control (Bryce *et al.*, 1994; Slutsker and Kachur, 2013). Yet with the vast burden of malaria falling on resource-constrained countries, a wide range of other stakeholders are typically involved in malaria decision-making at a national level, including non-governmental / civil society organisations (NGOs/CSOs), research agencies, donors, international implementing agencies and other government agencies. These agencies may have differing established strategies, expertise or ideas on how to respond to malaria, interacting with national bodies through formal and informal structures and ultimately influencing policy decisions and programmatic action.

These economic, global and domestic political realities raise questions about what forces are driving malaria control decision-making at the national level, and how various arrangements of stakeholders and financing shape strategic and programmatic decision-making beyond the policy and planning approaches of technical evidence review alone.

## Methods

This paper explores these issues through a qualitative analysis of in-depth interviews conducted as part of the evaluation process of the LINK programme. This evaluation had multiple objectives. One of these related more directly to evaluation of the utility and application of the country profiles and national risk maps provided to countries (Ghilardi *et al*., 2020). Another spoke more explicitly to the question of what ‘evidence use’ means from the programmatic perspective of NMCPs (Parkhurst *et al*., 2020) (results under review elsewhere). In this paper, we provide results from the evaluation objective which aimed to explore the political factors shaping malaria control decisions, outside of the simple review of technical evidence alone.

Qualitative interviews were conducted between April 2018 and July 2018 with a total of 177 stakeholders based in the Democratic Republic of Congo (DRC), Ghana, Kenya, Malawi, Mali, Sierra Leone, Uganda and in global agencies. Purposive sampling was undertaken to capture a range of perspectives of individuals involved in evidence provision, decision-making and implementation of malaria programmes. It included representatives of government agencies, non-governmental organizations, UN agencies and researchers—although the largest group of interviewees represented NMCPs (48 individuals). Table 1 provides a summary of interviewees by country and agency type represented.

**Table 1:** Summary of respondents

	Kenya	Malawi	DRC	Mali	Sierra Leone	Ghana	Uganda	Global level
NMCP	1	5	7	5	10	8	10	0
MoH (Health information, Policies, Prevention& Control/ Research)	1	0	2	1	2	3	1	0
MoH/ District level	0	0	0	0	2	2	3	0
Gov bodies (statistical office, Pharmaceutical bodies, medical supply)	1	0	0	3	3	1	0	0
UN agencies	3	1	2	3	3	3	3	9
Donors	1	3	4	2	2	3	3	0
NGOs/CSOs	4	5	10	7	3	8	4	2
Researchers	2	4	2	2	3	2	2	0
Total	14	18	27	23	28	30	26	11

The majority of interviews were conducted in the capital city of each included country, although some district-level interviews were conducted in Ghana, Sierra Leone and Uganda. Ethical approvals were obtained from relevant national bodies in each country along with the London School of Hygiene and Tropical Medicine, with informed consent obtained for individual interviews.

Stakeholder interviews followed an interview guide that aimed to elicit descriptions of the decision-making processes involved in malaria control (the Supplementary Interview Guide is available as an online supplementary file). This included broad policy themed questions aimed at providing insider descriptions of policymaking dynamics—including the roles of stakeholders and actors within and outside the MoH, and the steps and stages of malaria decision-making processes. It also included questions more specifically related to the remit of the LINK programme—such as stakeholder understandings of prioritization and targeting in malaria, and stakeholder views on the production and use of data for decision-making. A final set of questions were then asked to explore the factors affecting the specific use of maps and data from the LINK project and the related ‘Information for Malaria’ (INFORM) project (these data are analysed elsewhere).

Interviews were conducted in French and English. They were recorded, transcribed verbatim, translated (for French interviews) and coded both deductively—using an initial coding framework—and inductively—using content and thematic analysis, assisted with the NVivo 11 qualitative software package. The coding framework was organized on five initial levels: (1) evaluation of the LINK project; (2) data production, availability and access; (3) data use and data pathway; (4) political economy and decision-making structure; and (5) health system. Data for this paper were principally extracted from level the level four coding, although at times level 5 coded data provided useful insights. Once coded, data were subsequently analysed to categorize and identify themes that could be helpful in exploring the emergent findings in relation to factors other than provision of data or evidence that were important to shaping malaria policy choices. After initial coding of interviews, researchers identified novel emergent findings from the study, which led to further review of coded data to explore political factors shaping decisions in more depth. From the discussions on emergent findings and second round of data coding, it became clear that explanation for different emergent findings could benefit from classification and exploration along the ‘3I’s’ framework of *interests*, *ideas* and *institutions* (Hall, 1997; Béland, 2016) —which was then adopted as an analytical framework to support this process and structure results of this paper.

### Political economy insights through an analytical framework of the ‘3Is’

The initial impetus to the study was the inclusion of questions for stakeholders that could allow exploration of political economy factors that might be important to shape malaria policy decisions. As a starting point, a broad perspective on political economy was taken, in line with Bump and Reich (2013) who emphasize the approach as making ‘explicit recognition of the importance of both politics and economics in the distribution of resources (p. 124)’. However, political and economic relationships may manifest themselves in a number of different ways.

Smith *et al.* (2014) have argued that the 3I’s perspective can be particularly helpful to understand the politics of healthcare resource allocation, but they explain that its application has been limited in the health sector, despite its ubiquity in the broader political science literature. There are some examples, however, of its application to health issues in low- and middle-income countries—with Cliff *et al.* (2010) specifically analysing interests and ideas to understand the choice between two competing malaria strategies in Mozambique, South Africa and Zimbabwe.

Typically, the concept of *interests* is the most intuitive and widely recognized of the three, capturing how stakeholders pursue their personal or collective goals in the promotion of policy decisions. Stakeholder roles and authority was a major theme in the research interviews, as it was recognized that a large set of domestic and international actors can be involved in malaria control. Interest-based analysis can raise questions around how donor interests and pressures may clash with national autonomy and policy directions, as noted in other global health policy examples (Okuonzi and Macrae, 1995; Khan *et al.*, 2018), and thus in relation to malaria, it provided a lens of analysis to look more specifically at how the key stakeholders known to be involved in funding or advising national governments might have particular goals or priorities in relation to domestic perspectives.

Turning to *ideas*, Parsons (2002) defines these as ‘subjective claims about descriptions of the world, causal relationships, or the normative legitimacy of certain actions (p. 48)’. Thus ideas can be seen to represent collectively shared thoughts about what is the ‘right thing to do’ in relation to malaria control. This provided a second lens of analysis, to look at coded data so as to see whether or how collectively held ideas within the malaria control community might be shaping decisions, or if there could be tensions in ideas as well—such as whether ideas from outside (such as those of expert bodies) might somehow misalign with ideas on what was needed from the perspective of local stakeholders.

Lastly, *institutions* are seen to capture a range of more or less formally organized structures, rules, processes and norms that work to direct political behaviour and decisions (Peters, 2005) As such, they encompass the administrative elements of national programmes responding to malaria (i.e. NMCPs), as well as broader national policy structures and global health arrangements through which malaria decisions and actions are decided. This provided the third lens of analysis to look at data to explore the importance of formalized arrangements or structures within countries which would have developed as a result of, or in relation to, existing funding and technical advice systems—and which appear to play important roles in shaping malaria policy and intervention choices.

While some authors describe the 3I’s as ‘independent variables’ for political economy analysis, in as much as they are seen to determine policy outcomes (Hay, 2004) they are not typically assumed to be independent of one another. Rather it is recognized that they are interdependent and often mutually reinforcing. Scholars thus note how institutional structures may function to promote particular interests, or how institutionalized norms can prioritize particular ideas or logics that can drive policy action in ways other than the pursuit of the personal interests of stakeholders (March and Olsen, 1989; Peters, 2005). Taken together, the 3I’s approach was used to explore key dimensions of the political economy of malaria control that existed outside purely technocratic considerations, and outside the rational evaluation of evidence provided.

## Results

The themes explored in this paper draw out ways that features of the political economy of malaria may play out at national levels, to provide explanations of policy choices outside of those arising from a purely technical review of data or evidence. That said, it is worth starting by noting that our interviews did, in fact, find that a significant amount of decision-making at national levels involved technical planning choices informed by reviews of relevant epidemiological or related evidence. These captured decisions around which products to provide for malaria treatment, whether to begin an intervention, or where to provide an intervention. Specific examples mentioned included: whether to provide either two or three doses of sulphadoxine-pyrimethamine as a preventative dose (Malawi, Kenya); where to undertake spraying of insecticides (Ghana, Sierra Leone); whether to scale up other prevention efforts to national levels (Sierra Leone, Uganda) or have Long-Lasting Insecticidal Net (LLIN) coverage nationally (Uganda, DRC).

There were, however, several aspects of malaria decision-making that showed how decision-making and programmatic activity often went beyond technical considerations alone—with interviews exploring multiple political and economic factors that appeared to steer or direct these technical choices in ways beyond technical reviews of data and evidence. These insights are presented below structured along the conceptual division of the 3I’s.

### The competing *interests* of stakeholders

An important starting point to understanding the political realities of malaria control is to explore the interests of the multiple stakeholders involved. As noted, financing is primarily provided by international donor bodies and (to varying extents) from national governments. Interviews asked respondents to describe the role of different stakeholders involved in funding and deciding malaria control activities. Funding discussions focused on two of the largest bodies in particular—the US’ President’s Malaria Initiative (PMI) and the Global Fund for HIV, TB and Malaria (the Global Fund). Some mention was made of other donors at times, however, such as the UK Department for International Development, or UNICEF, but with less frequency. Interviews also asked for descriptions of the variety of stakeholders involved in planning and implementation of activities. From these explanations of stakeholders and their roles, several themes emerged in relation to the interests of the different actors, and how stakeholders pursuing their interests could affect the decisions and roles of national planning bodies.

### Donor vs local priorities

Donor funding, in and of itself, is recognized to have implications for local political agendas and priorities (Khan *et al.*, 2018). Indeed, the vertical funding of donors on disease-specific issues (through funding bodies such as the Global Fund and others), has for many years been critiqued for distorting national policies by creating powerful or well-resourced groups around a preferred topic (Biesma *et al.*, 2009). These critiques often centre on the relative priority that preferred topics of donor funding may achieve at national level (e.g. the importance placed on HIV/AIDS, TB or malaria in relation to other national health priorities). Yet there can also be intra-issue priority-setting dynamics in which the interests of donor agencies and local stakeholders play out.

Several interviews with current or former officials working within Ministry of Health programme offices noted how donor priorities within malaria control were not always aligned with local realities or needs. The Global Fund application processes, for instance, require countries to have an updated Malaria National Strategic Plan—from in which objectives and key activities are prioritized and funding requested through a country Global Fund concept note. One Malawian official, however, suggested that the Global Fund preferred to fund commodities, with little interest in funding behaviour change programmes needed to make them work:

*… commodities come first. Next is the nets. After the nets they look at other interventions. But amongst other interventions, BCC [behaviour change communication] comes last… when you give out those nets [they] are not used, so it’s a waste… you’re wasting resources… But unfortunately it’s not funded by Global fund, it’s pushed last* (MoH #6, Malawi).

It is worth noting that governments applying for Global Fund grants are meant to demonstrate some co-financing, so it is possible that the Global Fund focusses on certain key commodities, hoping the government will take up other activities. However, this feeling of neglect of other areas was identified in interviews from MoH officials. For instance, in DRC one former senior technical official lamented a lack of focus on prevention in supported programmes explaining: ‘…it's good to have the drugs, but if we do not attack things upstream, we do nothing’. Another DRC official explained that ‘partners’ (donors) come with pre-determined plans noting: ‘when a partner arrives, he has already made his budget and schedule, it's a little difficult to change (MoH #17, DRC)’.

In Kenya, a government official reflected on how donor finance can shape policy priorities by ensuring some health issues have stronger data:

*Let me give you a good example. HIV is the strongest because they can review, they can print tools and then they can disseminate. They have money…. TB as you can see, has as a team… because they could be able to fund it, partners came up… So the program money you think would be a lie to say that has no influence* (MoH #3, Kenya).

The importance of demonstrating the impact of the intervention was also seen to influence choices such as the selection of the indicator to use to monitoring/evaluating impact or the area chosen for a specific intervention. For instance, one development agency representative stated that ‘[o]ur main indicator for impact is mortality (Partner #4, Malawi)’; while another specifically explained that their decision to moving an IRS intervention to a different region was in order to have a larger impact (Parner #6, Mali).

### Coordination of stakeholders

Initial questions asking participants to describe stakeholders and their roles allowed identification of situations where priorities may differ between national and national actors. However, interviews also discussed the nature of planning and decision-making more broadly. From these discussions, another theme emerged in relation to stakeholder interests—specifically how non-state actors would pursue their own interests for intervention activity in ways that would fragment activities within a country. This could provide another challenge to the idea of comprehensively rational planning at a national level, and was recognized by a range of interviewees. One senior government technical officer in Mali gave as an example how within that country:

- The Global Fund, PSI (an international NGO), MEASURE (international USAID supported agency) and other local NGOs were active in the national concept note development;
- Service provision was provided by French and Spanish arms of Médecins Sans Frontières, (MSF) in different regions (in a humanitarian response);
- USAID was providing support via PMI and MEASURE;
- UNICEF was ‘providing’ chemo-prevention [likely meaning supporting or funding in this setting];
- WHO was undertaking epidemiological surveillance;
- Canada was providing aid to the Ministry; and
- China was providing drugs (MoH # 1, Mali);

Such situations may not in fact be unusual in the health sector. Indeed, our team of researchers noted that Mali was not unique in this regard, with all countries showing a high number of stakeholders, fragmentation of activities and multiple stakeholders involved in Global Fund proposal writing. Other interviews conducted with international agency staff based in country also reflected on the multitude of non-state actors working on malaria programmatic action: noting how international NGOs might have local branches undertaking activities with the NMCP (Partner #1, Sierra Leone) or how international NGOs might pilot new WHO recommendations to then pass on to government (Partner #22, Uganda).

Almost all non-government stakeholders interviewed said that they follow the National Malaria Strategy, they are working under the NMCP, and they participate in the technical working groups (TWGs) aimed to make strategic decisions and coordinate activities—indicating some level of adhering to the interests of the recipient nation. However, a critical political economy perspective needs to at least recognize that national strategic plans and coordinating bodies may not necessarily perfect reflect national priorities or local accountability—especially if their own origins may have been influenced by outside stakeholders. So, e.g. TWGs regularly include membership of non-state actors. National plans are also often developed with input from international actors, with one analysis by Andrada *et al.* (2019) of malaria strategic plans in 22 countries concluding that: ‘Most [national malaria] targets were set according to global goals rather than the individual country’s previous achievements and limitations’. This may in part be due to an expectation that national strategies should align with the global technical strategy—thereby limiting the ‘room for manoeuvre’ (see Clay and Schaffer, 1984) for a local government to truly shape priorities in a locally defined way.

Our findings further found that non-state actors often did not take direction on where to work or which activities to undertake from state governments per se, even if activities fell under the broad heading of the national strategy. The reality of such a wide number of stakeholders involved, with differing resources and potential influence, meant that the coordination or management of stakeholders ended up as a key responsibility of NMCPs.

The importance of this coordinating role for NMCPs was particularly apparent in response to the realities that partner agencies would often strategically divide up countries for implementation of activities—a situation mentioned by both government officials and international partner agency officials in multiple countries. In Uganda, for instance, when talking about the organization of one specific programmatic strategy (village health teams—a form of community health workers), one MoH official explained:

*…it’s not uniform in all the country and being project driven we have some funders like Malaria Consortium they go and fund in 18 district, UNICEF goes to 18 districts, USAID goes to 18 districts…* (MoH #5, Uganda).

In DRC, there were also reports of specific gaps in supply coverage due to the lack of resources combined with the division of responsibility between actors. According to a respondent from an international partner agency:

*Before, there was almost no sharing of information between different stakeholders around the NMCP, so you can easily find in one province an area that has overstock convenience for malaria control and another right next to it out of stock* (Partner #25, DRC).

It was further explained that the NMCP now works to analyse stocks of supplies nationally, to overcome this problem. This respondent noted how the NMCP acts to try to ‘rationalise’ the situation—attempting to get international actors to agree which areas they would each focus on in a more coordinated manner.

Finally, developing and ensuring adherence to a single national plan served as another key strategy for partner coordination as well. As explained by a government official in Kenya:

*Even the partners when they’re putting money, they’re putting money on what the strategy has said. Whether it’s global fund, whether it’s USAID, whether it’s DFID. It has to be what is in the [NMCP/national] strategy* (MoH #1, Kenya).

Thus, national programme actors regularly saw their interests lying in harmonizing, coordinating, managing and aligning other groups activities, rather than simply planning and directing programme actions based on traditional technocratic considerations.

### Clashing *ideas*—universal coverage vs targeting

While the interests of stakeholders help to understand some of the realities of policymaking at a country level, interviews also were open to allowing respondents to describe why or how decisions were made, with probing and follow-up questions often asking for explanations of this nature. These data allowed further analysis along a broad theme of ideas, to analyse data discursively to see how problems and solutions to malaria control were constructed and understood. In our study countries, there were clearly well-established ideas about how reviews of technical evidence and WHO guidelines were important to integrate into national plans and action—concepts that would align well with expectations of rational technocratic planning approaches. However, we also identified other instances where established global ideas could lead to challenges at the local level.

The most notable of these was how the language around a globally embraced idea of ‘universal coverage’ for malaria control could be in tension with locally experienced logics of priority setting and targeting of activities. Priority setting is a commonly followed process used when decision-makers have set budgets with which to maximize health gains (Green, 2007). Yet Smith *et al.* (2014) have argued that, in reality, much priority setting in health has been driven by historical and political concerns, rather than purely technical evaluations. In the international malaria community, calls for ‘universal coverage’ have been an important rallying cry, embedded in global reports [see chapter 3 in WHO (2011)].

The concept appears to date back to 2005 when the Roll Back Malaria Partnership, further endorsed by the World Health Assembly Resolution WHA 58.2 (Kiszewski *et al.*, 2007), encouraged countries to implement a two-pronged malaria control approach: firstly a ‘Scale Up for Impact’ (SUFI) strategy, and secondly maintenance of universal coverage (Campbell, 2008; Roll Back Malaria Partnership, 2008). SUFI was enshrined in the Global Malaria Action Plan developed in 2008 which was embedded in many NMCPs (Steketee and Campbell, 2010). This plan has since been superseded by newer global guidance, but at the time of our fieldwork, the associated idea of universal coverage was still prevalent, and could be seen to lead to some implicit contradictions. For example, a respondent from an international organization in Mali stated:

*Well, until proven otherwise I think we have to go with [universal coverage of interventions] because in Mali, the whole population is at risk. It is necessary to go with the universal [coverage of] interventions.*

*[INTERVIEWER: Even in the North of the country, it is not low prevalence?]*

*Yes, yes. But in general terms in Mali, the whole population is at risk* (Partner #8, Mali).

In another case a Ghanaian government official explained ‘…everything that we do targets the whole population…Because it’s a national, malaria is a national concern, a public health concern’. Yet the individual continued by stating *‘*we segment audiences for various interventions. But even with that… we can’t single out an individual or a particular group to reach out (MoH #6, Ghana)’.

Other times, however, national government officials more explicitly saw the push for universalism as problematic. As one Ghanaian official explained:

*…it shouldn’t be like one size fit all. If we’re targeting interventions we should be able to tie it to the prevalence and epidemiological situation… You don’t go distributing bad nets throughout the country… We need to look at those who have lower burden and those whose burden are higher*—*if you want to deploy indoor residual spraying, we need to know where to do it, and where not to do it* (MoH #4, Ghana).

A similar view was expressed by a researcher in Uganda:

…*we need to be smart at how we target our limited resources because you know why should you put the same intensity of intervention in an area that has less than 1% and another one has 50% parasitaemia…* (Researcher #4, Uganda).

These tensions reflect the obvious need to allocate limited resources within a global rhetoric that presses for universal coverage—but which may remain unclear or unspecified when local officials attempt to apply the concept in practice. National programmes are beholden to international funding bodies, and those bodies have used the ‘universal coverage’ idea to justify higher levels of global finance. Yet while many NMCPs do wish to cover the entire population, in the realities of national settings, targeting high-risk groups was often seen as an essential reality.

### *Institutionalization* of processes and systems

The final element of the 3I’s framework focuses on institutions—the structures, processes or norms formalized within national programmes that can influence malaria control decisions and actions. In our analysis, we identified two emergent examples that pointed to the importance of institutionalized systems playing important roles in shaping malaria decisions, or decision-making, over time. The first was how the influence over policy and action by non-state actors (the interests of stakeholders) could become institutionalized into malaria planning systems. The second was how the established structures of malaria response risked undermining development of local capacity in ways that could hinder the idea of a country simply assessing evidence in a rational technical planning manner.

The first of these reflects Harrison’s (2001) idea of ‘post-conditionality’—the formalization of systems of planning and decision-making in aid-dependent countries that work to embed mechanisms of policy influence beyond simple conditions applied to aid financing. The aforementioned normalization of the process of dividing countries up for donor activities, or the long-term acceptance of the high number of implementing partners, could be seen to reflect such institutionalization. As already discussed, this can result in NMCPs serving more as coordinator or manager of activities, rather than directly deciding or controlling activities.

Our case studies provided further details, however, on how policy influence could be institutionalized. For example, in Uganda, and Mali there was the establishment of a Ministry of Health position under the NMCP to serve as ‘coordinator of the Global Fund’—providing further legitimacy to the coordination function and reliance on outside actors. Another way that international influence could be formalized into planning processes, however, was through the reliance on TWGs. TWGs were often established as the key bodies reviewing technical evidence and directing interventions, yet they typically have formal membership from researchers, programme partners, and implementing bodies alike. As noted above, they are often presented as the mechanism through which stakeholders can be coordinated, yet in some countries, TWGs themselves are set up on the recommendation of international bodies such as the WHO (2005). They further typically contain representatives of international partners, raising questions about how much they can be taken to reflect purely local priorities or stewardship.

In Kenya, a researcher interviewed explained:

*it is the technical working groups that meet and discuss specific issues to do with policy direction and implementation … Then the technical working group will feed into the Malaria Inter-Agency Coordinating Committee… These working groups are the key drivers of malaria control* (Researcher #10, Kenya).

Similarly in Uganda, TWG ‘sub-groups’ were set up to direct more specific activities. One government official described the TWG for malaria in pregnancy:

*its attended by all key stakeholders like for example all implementing partners that are carrying out activities for Malaria in Pregnancy prevention sends in their representatives to attend that meeting WHO is also on the lead… we check performance we do update ourselves and do planning on what we want to do in the next quarter* (MoH #8, Uganda).

In the Kenyan case, others explained how influence could play out through the TWG. An NGO representative explained:

*I think there are certainly some partners who are more influential when it comes to making decisions. One, because of, I think, the contribution they bring to the table in terms of technical knowledge, but also the resources they bring to the table. So I mean for example, WHO is very influential, you know they have been given that space of [technical assistance] and the ministry respects that … DFID has not been influential at all in the malaria's phase in this country because although they have put in a lot of resources, nobody sits from DFID in the TWGs … PMI are on the table influencing decisions including decisions to do with the Global Fund. So they also influence how Global Fund resources [will be used]…* (Partner #6, Kenya).

Finally, in Malawi a sentiment was expressed that TWGs were now supporting the NMCP in key ways: ‘it’s basically now that instead of the program just doing things on their own but it’s basically the involvement of other partners throughout these technical working groups (Partner #14, Malawi)’.

Thus while there may be good reasons to establish TWGs in situations involving a range of funding bodies and stakeholder service provision, their establishment alters the decision-making institutional space—formalizing and embedding the influence non-state bodies can have over national malaria policy.

### Local capacity challenges

The final institutional theme identified raised in our interviews captured concerns about local capacity in the country. While it is often argued that low-income settings face capacity challenges, it was also clear that systems were routinely followed which allocated planning elements to external bodies, or which maintained a more limited role of national bodies. As such, key processes and activities that were institutionalized within the country could themselves perpetuate the idea, or even the continuation, of local capacity limitations—e.g. if the routine bypassing of local stakeholders or use of alternative systems of planning removes opportunities for local capacity to develop. For example, in Malawi, a researcher explained that a change in national drug treatment policy could not come from within the country due to the lack of a relevant local body that could undertake data analysis:

*that change in policy is not because Malawi … had a coordinated way of saying this is what we want, but what had happened is studies were conducted in Malawi and outside Malawi and then later data was sent to WHO. And it was WHO that came with the final decision … even … the data that were collected in Malawi there was no particular platform that could synthesize those data …* (Researcher #5, Malawi).

Alternatively, in Uganda, a government official noted their lack of ability to align partners:

*having this small structure is not enough … if you are empowered and technical you would be able to control the partner if you like that partner to do but since you are at a low level the aligning then becomes difficult they are the ones with the money you see its difficult so you find the system becomes weak because you cannot control those* (MoH #6, Uganda).

Finally, an interviewee in Sierra Leone described the limited role taken by the government when a programme for preventative treatment in infants was being developed, explaining how it was WHO involved in piloting, UNICEF in scaling up and ICAP (an international NGO linked to Colombia University) who did the monitoring and evaluation—while the Ministry of Health managed training (Partner #19, Sierra Leone).

## Discussion

Our analysis utilized the perspective of interests, ideas and institutions to explore the political and economic realities of malaria control that can influence policy and practice at a national level, beyond reviews of technical evidence alone. Many of the issues identified appeared to be in response to the specific global and national contexts of malaria control, however. In particular, the funding landscape is dominated by a small number of key agencies (with the Global Fund particularly dominant), leading to significant scope for influence; while implementation of malaria control is typified by a large number of stakeholders, leading to issues in coordination and clarity in government roles. Global malaria efforts further are dominated by a relatively circumscribed body of experts shaping global thinking and directions in malaria policy which can then be disseminated to countries.

The heavy reliance on the Global Fund—a globally pooled source of funding—could arguably make countries more susceptible to funding body interests, as it places countries in a weaker position than might be the case when there are multiple donors with which a country can negotiate. Mosley *et al.* (1995), for instance, have described conditional aid lending (by the World Bank) as a ‘game’ situation whereby both parties are bargaining to maximize their interests, and give up the least. More recently, Manning (2006) and Sato *et al.* (2011) have both argued that proliferation of donors can give countries greater options or improve outcomes; and in one specific health example, Han and Koenig-Archibugi (2015) found that a multiplicity of donors in aid-recipient countries was correlated with lower child mortality rates then when fewer donors were active (this result held unto a point, however, after which increased diversity of donors appeared to raise mortality rates again).

Multiple lenders can thus potentially provide more options, and more appropriate or effective options in cases where single lenders may have little reason to consider alternative approaches. Single lender situations also can place national programmes in awkward positions if the interests of key donor agencies simply differ from national priorities as well (regardless of effectiveness). Donor agencies may also have dominant ideas around how to address particular health issues—as seen in one analysis by Steele (2011) who found health donors particularly favouring direct prevention and treatment activities over broader infrastructure, research or educational efforts.

The funding landscape thus heavily shapes which interests of what stakeholders are brought to bear on malaria control efforts, as well as which ideas are promoted. The nature of the international expert network guiding global malaria action can further play important roles in shaping collective ideas—such as the importance of universal coverage, promoted globally as part of overarching efforts to scale up malaria control. Such ideas may be useful for global agenda setting, but risk contrasting with local realities, where technical planning approaches will typically mean officials need to consider the most effective use of limited resources. Indeed, the idea of ‘universal coverage’ appears to mean different things to different people—from ‘everyone, everywhere, irrespective of epidemiology being covered’; to ‘those most vulnerable having universal access’ (such as under 5’s or pregnant women); to ‘those in particular epidemiological strata having 100% coverage of specific preventative strategies’. Although, universal access to diagnosis and treatment does appear to have a common interpretation of testing for all, outside of this, ‘universal coverage’ is still an idea that needs further clarity and nuancing, especially if it is being used to justify funding and shape policy and practice.

While there may be only a limited number of funders and global health expert networks, the contrasting large number of non-state stakeholders involved in implementation of activities was found to result in additional challenges and impacts on national bodies. NMPCs often saw their roles as involving management and coordination of non-government stakeholders, rather than more typical technical planning or service provision. In our study, the institutionalization of particular modes of planning and responding to malaria, in relation to these dynamics, had further implications for national control over policy agendas or capacity to respond. These findings align with observations of other more politicized global health issues as well. Work looking at HIV/AIDS, for instance, has argued that reliance on multiple global actors who delegate implementation to distant bodies can undermine governance systems in local settings, with misalignment between donor bodies and local priorities or changing expectations by the public about what social services the government can or should provide (Dionne, 2017). While the present study did not explore beyond the malaria control sector, it reinforces concerns about how local needs and priorities may be affected when international stakeholder influence is institutionalized into public health responses.

## Conclusion

NMCPs sit at the heart of malaria responses in countries. They are mandated by Ministries of Health to take responsibility for malaria control, and typically staffed by technical staff—public health and infectious disease officials who strive to use data and evidence to select the most effective package of activities possible. Yet this highly technocratic exercise cannot be divorced from the contextual realities of the local policy environments. Political economy factors provide a set of challenges (and opportunities) for action, placing planners in a decision-making tunnel that constrains their actions in certain dimensions. Even if provided with robust data and epidemiological evidence that could guide planning activities, NMCPs must undertake malaria control efforts within the established systems and structures that connect sources of funding, implementing agencies and technical advice to national systems.

This paper has analysed qualitative data that aimed to investigate the stakeholders and processes involved in malaria planning from an explicitly political perspective. The 3I’s of ideas, interests and institutions was applied to the data to provide conceptual categories for thematic analysis. Interests were explored in relation to the multiple stakeholders involved in funding and implementing malaria control efforts, highlighting how donor priorities may not align with local needs per se, and the coordination challenges this can place on local authorities. Ideas, on the other hand, permitted deeper exploration of how dominant concepts about the ‘right thing to do’ in malaria control could lead to challenges at times—such as when global concepts of universal coverage had to be applied within constrained local contextual realities. Finally, an institutional lens allowed consideration of how the systems and structures constructed to plan and implement national responses could both reflect, and embed, political and economic influence from non-state actors.

Malaria remains one of the leading contributors to premature mortality in the world and a critical global health priority. While technical interventions including drugs, mosquito nets and insecticide spraying remain central to malaria control, policy and programmatic action must be made at country level to ensure an effective use of those strategies to achieve success. Technical as it may seem, the practical realities of malaria control evolve within specific contexts of political and economic forces that shape both what is possible what remains a challenge, and which interests shape national responses to the disease.

## Supplementary data

Supplementary data are available at *Health Policy and Planning* online.

## Funding

The authors were supported by the LINK programme, funded by UK aid from the Department for International Development (DFID) for the *Strengthening the use of data for malaria decision-making in Africa* project; however, the views expressed do not necessarily reflect the UK government’s official policies. RWS is supported as a Wellcome Trust Principal Fellow [grant numbers #103602 and 212176].

## Supplementary Material

czaa166_Supplementary_Data
