# Supplementary material for: Competing interests, clashing ideas and institutionalizing influence: insights into the political economy of malaria control from seven African countries
Source: Health Policy Plan. 2020 Dec 14;36(1):35–44. doi: 10.1093/heapol/czaa166 (PMC7938496; doi:10.1093/heapol/czaa166)
Supplement: czaa166_Supplementary_Data [file czaa166_supplementary_data.zip › Interview_guide_general.docx]

**INTERVIEW GUIDE**

#### Stakeholders: Staff of the National Malaria Programme

**Main objectives:**

1. To describe the national level malaria policy decision-making architecture, policy and political context, in country X (Political Economy of Decision Making (PE4D)
2. To assess the use of the malaria epidemiological profiles generated by the LINK project, and other data, in decision-making for malaria control in country X. (Data use)
3. To explore assess the mechanisms through, and context within which, the LINK project, and other data, is used in malaria control decision-making in country X. (Data pathways)

**Synthesis:**

| **National Malaria Programme** |  |
| --- | --- |
| **ALL** | - **Role, data and information used in their work** - **Which decisions on prioritisation and targeting they make if any** - **Responsible for national policy/strategy docs – NMS, GF applications, MPR, national guidelines etc – who writes them, who reviews them, who signs off on them** - **Role of partners/ MoH / Donors** - **Knowledge of Link ? Point of view on Link, use of LINK data – for what, when – all examples** |
| **Director/Head/Coordinator of NMP** | - Decisions on prioritisation and targeting – process, data used, why, guidance gaps, examples. - TWGs – which have, chaired by who, their role, role in decision making on prioritisation and targeting - Divisions of NMP – their role in decision making – prioritisation and targeting – how do they link with TWGs - Role of the Malaria Interagency Coordinating Committee (MICC) - Does he Identifying himself as the person who makes decision in malaria or he identifying stakeholders above him ? Which are they? Which are the challenges? |
| **NMP focal person for surveillance, M&E and operational research** | - Source of data, use of data, research management - who uses the data – how is it presented to them – processes for this – feedback loops – prioritisation and targeting, who are the decision makers - opinion on the MIS/DHS and MICS (Multiple Indicator Cluster Survey) - Links with TWGs and MICC - How they do monitoring? Evaluation? - How do they identify Research Gaps? How do they work with the research community? |
| Focal point division of NMP for case management | - Source of data, use of data for coverage of intervention, identification of gaps - Collection of routine data - Utility of data on coverage and interventions in the profile? |
| **Focal point division of NMP for vector control** | - Main interventions and modality to deliver interventions - Modality of quantification of nets - How and where they define IRS interventions? - Role in prioritisation and targeting decisions, examples, other decisions, links with TWGs, and MICC - Use of data which data, why, gaps |
| Focal point division of NMP for malaria in pregnancy | - Role in prioritisation and targeting decisions, examples, other decisions, links with TWGs, and MICC - Use of data which data, why, gaps, |
| **Malaria Interagency Coordinating Committee Chair** | - Role in prioritisation and targeting decisions, examples, other decisions, links with NMP and TWGs, who chairs - Use of data which data, why, gaps, |
| NMP Financial Department (Planning, Finance and accounting) | - Domestic and International expenditures |
| **TWG Chair (for each NMP TWG)** | - Role in prioritisation and targeting decisions, examples, other decisions, links with NMP, and MICC - Role in national document writing – NMS, GF applications, MPRs, national guidelines etc - Use of data which data, why, gaps, - Insecticide resistance management (vector control TXG) - Role of partners - Knowledge of Link ? Point of view on Link, use of LINK data – for what, when – all examples |

**Full:**

| 1. **Background to interviewee** | ALL |
| --- | --- |
| - 1. Could you tell me your name and your current role? |  |
| - 1. How long have you been working in the same position? / Where have you been working before? |  |
| 1. **Decision making process and structure** |  |
| **Inside the MoH (internal structure)** | ALL (according to each role) |
| - 1. What is the role of your Department in the planning/policy OR research OR financing in malaria? |  |
| - - 1. What is your particular (individual) role within the process? |  |
| - - 1. What contact do you have with other actors in the process? |  |
| - 1. Which other departments and Ministries are involved in this process? |  |
| - - 1. How are they involved and at what stage?/ Which role? |  |
| - 1. Can you tell me about how malaria planning currently takes place ? Which are the main outcomes and products of the planning? (NMS, Malaria Policies, NMR, Guidelines, Annual Plans…)? |  |
| - - 1. What are the key steps in this process? |  |
| - - 1. Who are the key actors in this process? |  |
| - 1. What do you define as prioritisation and targeting in malaria?   2. Do you think it is necessary to prioritise and targeting malaria interventions in your country?   3. Do you think that decisions on prioritisation and targeting of interventions for malaria control are taken within the MoH?   4. If yes, decisions on prioritisation and targeting are made on the base of which type of data?   (e.g. Having a limited budget, how the MoH decide which intervention and modality of delivery the intervention to prioritise? Having a limited budget, how the MoH decide which areas or populations need to be targeted?) | Head/ Finance |
| - 1. It is possible to know the expenditure for malaria control by each year? It is possible by type of intervention and geographical area? How do you prioritise where and in what to invest money?) | Finance |
| **Outside the MoH/ at national level** |  |
| - 1. What are the key organisations with authority to make decisions on Malaria at national level (if any in addition to the MoH)? | Head |
| - 1. Can you describe the mechanisms of collaboration between the MoH and the NMP? And the Interagency Coordinating Committee? | Head |
| - 1. What are the official agencies/offices that gather, synthesise, and provide evidence to inform key office in the MoH? | Head |
| - 1. Outside the MoH there are some actors more influential than others in the policy/planning process? | Head |
| 1. **Production & access of data (bdg, expenditure, implementation data, etc..)** | ALL |
| 3.1 What are the main purposes for which *you need data in your current role?* |  |
| 3.2 What decisions do you have to make in your role for which you need data? |  |
| 3.3 Is the data that you need generally available to you? |  |
| 3.4 What is available? What isn’t available? |  |
| 3.5 What are the strength and limitation of each source of data you are using? |  |
| 3.1 Which data and/or information do you need in order to develop your work? |  |
| 3.2 Does your department/office collects data on malaria? on which aspect of malaria (incidence, morbidity, insecticide resistance, etc…)? |  |
| 3.3 Which type of data are you (personally) and your organisation using for malaria control? In any, which data for prioritisation and targeting interventions? |  |
| 3.4 What are the main sources from which you access data that you use in your current role? (Try to get information on at least 3 sources) |  |
| - 1. Are there any official bodies tasked with evidence review/synthesis inside or outside the MoH? |  |
| **4. Production & Use of data (including use for prioritisation and targeting)** |  |
| - 1. What are the main channels through which Malaria knowledge transfer occurs (from the data/ research to the MoH and to your department) ? | Head/ research |
| - - 1. How could these mechanisms be improved? | Head/ research |
| 4.2 Do you think evidence/data could be used more effectively? | Research |
| - 1. To what extent the WHO recommendations are utilised in Malaria planning and control? | Head/ Policies |
| - 1. Are regulations that you are following on prioritisation and targeting? | Regulation |
| - 1. How you manage insecticide resistance? Which data are you using to manage insecticide resistance? |  |
| **5**. **Routine data** | Data/ head |
| - 1. In what ways do you use routine data from the Health Management Information System? |  |
| - - 1. Are there other ways in which you would like to be able to use it? |  |
| - - 1. If so, what are the barriers to this? |  |
| - - 1. Are there ways in which you think the HMIS could or should be improved? Do you have suggestions on how to do this? |  |
|  |  |
| **5. LINK profiles & tools** | ALL the first question (then only if they are familiar) |
| 5.1 Have you been able to access the epidemiological profiles produced by the LINK project? |  |
| 5.2 From where did you access the profile? |  |
| - Did you have any problems accessing the profile from this place? |  |
| - Where else is the profile available as far as you know? |  |
| - Which of these places that the profile is available is accessible to those who need it? And which are not? |  |
| - Why? |  |
| 5.3 Is there anywhere else or any other means by which you think the profile should be made available? Where? Why? And for who? |  |
| 5.4 Have the epidemiological profiles produced by the INFORM/ LINK programme been useful to you? |  |
| - How? |  |
| - And why? |  |
| - 1. Which data in the profiles were particularly useful and why? For what purpose did you use these data? |  |
| - 1. Which maps were the most useful (and which were not)? For what purpose did you use these maps? |  |
| - 1. Have you used these data in any other way, even if not directly? |  |
| - 1. Did you feel confident in using LINK/ INFORM data? |  |
| - If not why? |  |
| - 1. Was there anything or anyone that you would say encouraged you to use these data? |  |
| - 1. Were there any barriers that you had to overcome in order to use these data? What barriers? Why were they there? And how did you overcome them? |  |
| - 1. Were there any barriers to your using the data in the profiles that you were not able to overcome? |  |
| 1. **Data Knowledge Gaps / Research** | Research |
| - 1. In your opinion, which are the main knowledge gaps on the production and use of data for malaria control at national level inside the MoH? (e.g. having robust routine data or robust survey, specify….) |  |
| - 1. Who are the main stakeholders on malaria research in the country? |  |
| - 1. How research is used for prioritisation and targeting? |  |
| - 1. In particular, how you are developing research on insecticide resistance? |  |
|  |  |
| 1. **Additional information** | ALL |
| 5.1 Is there anything else you think is important for us to know? |  |
| 5.2 Is there anything we have missed out? |  |
| 5.3 Are there any documents you could recommend that might be interesting to this study? |  |
| 5.4 Who else would be good to talk to about this? |  |
| 5.5 Do you have any questions for us? |  |
