# Supplementary material for: Competing interests, clashing ideas and institutionalizing influence: insights into the political economy of malaria control from seven African countries
Source: Health Policy Plan. 2020 Dec 14;36(1):35–44. doi: 10.1093/heapol/czaa166 (PMC7938496; doi:10.1093/heapol/czaa166)
Supplement: czaa166_Supplementary_Data [file czaa166_supplementary_data.zip › Table 1.docx]

|  | *Kenya* | *Malawi* | *DRC* | *Mali* | *Sierra Leone* | *Ghana* | *Uganda* | *Global level* |
| --- | --- | --- | --- | --- | --- | --- | --- | --- |
| *NMCP* | 1 | 5 | 7 | 5 | 10 | 8 | 10 | 0 |
| *MoH (Health information, Policies, Prevention& Control/ Research)* | 1 | 0 | 2 | 1 | 2 | 3 | 1 | 0 |
| *MoH/ District level* | 0 | 0 | 0 | 0 | 2 | 2 | 3 | 0 |
| *Gov bodies (statistical office, Pharmaceutical bodies, medical supply)* | 1 | 0 | 0 | 3 | 3 | 1 | 0 | 0 |
| *UN agencies* | 3 | 1 | 2 | 3 | 3 | 3 | 3 | 9 |
| *Donors* | 1 | 3 | 4 | 2 | 2 | 3 | 3 | 0 |
| *NGOs/ CSOs* | 4 | 5 | 10 | 7 | 3 | 8 | 4 | 2 |
| *Researchers* | 2 | 4 | 2 | 2 | 3 | 2 | 2 | 0 |
| *Total* | 14 | 18 | 27 | 23 | 28 | 30 | 26 | 11 |

Table 1 – Summary of respondents
